# Distribution of carbapenem resistant *Acinetobacter Baumannii* and *Pseudomonas aeruginosa* and ESBL-producing organisms colonization among intensive care patients

**DOI:** 10.1186/1753-6561-5-S6-P293

**Published:** 2011-06-29

**Authors:** P Santanirand, K Malathum, T Chadlane, W Laolerd

**Affiliations:** 1 Pathology, Faculty of Medicine, Ramathibodi Hospital, Mahidol University, Bangkok, Thailand; 2 Medicine, Faculty of Medicine, Ramathibodi Hospital, Mahidol University, Bangkok, Thailand

## Introduction / objectives

Infection with carbapenem resistant *A. baumannii* (CR-*AB*)and *P. aeruginosa* (CR-*PS*) as well as ESBL-producing organisms have caused high rate of mortality. This project was aimed to survey the prevalence of drug resistant organisms colonization in critical care patients as a part of infection control program.

## Methods

Perianal swab, urine (from Foley’s catheter) and endotracheal catheter (ET) were collected from patients who were admitted to intensive care units of a 1000-bed hospital. Isolation of CR-*AB* and CR-*PS* was using EMB agar containing 12 ug/ml of either imipenem or meropenem. The MacConkey agar containing 1 ug/ml of cefotaxime was used for screening of ESBL-producing organisms. The MIC of these organisms was performed using THANF customised panel (Sensititre, UK).

## Results

A total of 81 isolates was detected from 39 patients. There were 53, 22 and 6 isolates of ESBL-producing organisms, CR-*AB* and CR-*PS*, respectively. Perianal was found to be the most common site for colonisation with ESBL-producing organisms (45/60 isolates) while 8 of 14 isolates from ET were CR-*AB*. All CR-*AB* isolates resisted to nearly all tested antibiotics. However, all isolates were susceptible to colistin and tigecycline with the MIC90 at ≤1 and 1 ug/ml, respectively. In contrast, the ESBL-producing organisms remained susceptible to all tested carbapenems. Nevertheless, 68% of these isolates resisted to fluoroquinolones.

## Conclusion

This project is a part of implementation of hospital-acquired infection control policy. The data demonstrated the existing of various multiple-drugs resistant organisms in critical care patients which would be a challenging task for infectious control.

## Disclosure of interest

None declared.

